# Atypical presentation of COVID-19 in a patient with type 2 diabetes at an urban primary care facility in Accra, Ghana

**DOI:** 10.4314/gmj.v54i4s.19

**Published:** 2020-12

**Authors:** Roberta Lamptey, Stephen T Engmann ST, Boateng Asante, Ernest Yorke, Yaw B Mensah, Samuel K Akoriyea, Christian Owoo, Henry J Lawson

**Affiliations:** 1 Family Medicine Department, Korle Bu Teaching Hospital, Accra Ghana; 2 Department of Community Health, University of Ghana Medical School, University of Ghana, Accra, Ghana; 3 Manna Mission Hospital, Accra, Ghana; 4 Ghana College of Physicians and Surgeons, Ridge, Accra, Ghana; 5 Department of Medicine & Therapeutics, University of Ghana Medical School, University of Ghana, Accra, Ghana; Department of Radiology, University of Ghana Medical School University of Ghana, Accra, Ghana; 7 Institutional Care Division, Ghana Health Service, Accra, Ghana; 8 Department of Anaesthesia, University of Ghana Medical School, University of Ghana, Accra, Ghana

**Keywords:** COVID-19, diabetes, primary care, low resource country, urban

## Abstract

This is a case report of a 55-year-old man with Type 2 Diabetes Mellitus who presented with progressive breathlessness, chest pain and hyperglycaemia. An initial impression of a chest infection was made. Management was initiated with antibiotics, but this was unsuccessful, and he continued to desaturate. A screen for Coronavirus Disease of 2019 (COVID-19) returned positive. There was no prodrome of fever or flu-like illness or known contact with a patient known to have COVID-19. This case is instructive as he didn't fit the typical case definition for suspected COVID-19. There is significant community spread in Ghana, therefore COVID-19 should be a differential diagnosis in patients who present with hyperglycaemia and respiratory symptoms in the absence of a febrile illness. Primary care doctors must have a high index of suspicion in cases of significant hyperglycaemia and inability to maintain oxygen saturation. Patients known to have diabetes and those not known to have diabetes may develop hyperglycaemia subsequent to COVID-19. A high index of suspicion is crucial for early identification, notification for testing, isolation, treatment, contact tracing and possible referral or coordination of care with other specialists. Early identification will protect healthcare workers and patients alike from cross-infection.

## Introduction

COVID-19 is a viral infection caused by the novel severe acute respiratory syndrome coronavirus 2 (SARS-COV-2). It was first reported in Wuhan, China in December 2019.^1^ In March 2020, it was declared a global pandemic by the World Health Organization (WHO).^2^ The average incubation period is six to eight days. This is followed by 1 to 2 weeks of symptomatic disease. The typical symptoms are fever, cough, myalgia, sore throat, fatigue, and malaise. Breathlessness is related to viral pneumonia and can lead to respiratory failure.^3^–^5^

The clinical presentation of COVID-19 ranges from an asymptomatic state to a severe acute respiratory disease.^6^ In severe COVID-19, chest imaging shows characteristic features which include ground glass opacities, consolidation, pulmonary nodules, fibrotic streaks and septal thickening. These changes tend to be more prominent in the peripheral and inferior aspects of the lungs. Chest imaging features tend to vary with the duration of illness and could be normal when taken early in the course of the disease.^7^,^8^

Diabetes mellitus is a predictor of morbidity and mortality in COVID-19.^9^ Person's living with type 2 diabetes mellitus (T2DM) are not at increased risk for getting infected with SARS-COV-2, but when they get infected, they are at increased risk for severer forms of the disease leading to prolongation of recovery time.^10^,^11^

Ghana had recorded 35,142 cases of COVID-19 as at 30^th^ July, 2020, with 31,286 discharges/recoveries and 175 deaths.^12^ About one-fifth of persons with COVID-19 disease are asymptomatic from the time of exposure to the time of admission. Furthermore, for those with mild symptoms, the most common symptoms are cough and hyposmia/anosmia.^13^ Asymptomatic carriers of COVID-19 have contributed to the challenges and the difficulty of prevention of transmission as well as prompt management of the disease.^14^

## Case Report

A 55-year-old male known to have T2DM for 5 years, presented to the Emergency Unit of an urban primary care facility in Accra, Ghana on 11^th^ July 2020 with complains of central chest pain and breathlessness on exertion of a week's duration. He was well until seven days prior to presentation when he developed gradual onset of difficulty in breathing and chest pain on exertion. The chest pain was non-radiating, moderate in severity, pressurelike and relieved with rest. He also admitted to easy fatigability but there was no associated fever, cough, rhinorrhea, sore throat, anosmia, ageusia, excessive sneezing, palpitation, orthopnea, paroxysmal nocturnal dyspnea, pedal oedema, early morning facial puffiness nor dizziness. He was not known to have hypertension or other chronic illness and he did not smoke. There was no history of confirmed COVID-19 contact. He was married with three children. His treatment regime included metformin 1g twice daily and glibenclamide 5mg twice daily, but he had not been compliant with his medications.

On presentation, the patient was afebrile (temperature 36.4°C), weighed 62.6 kg with oxygen saturation of 99% on room air. On examination of the cardiovascular system, his peripheral pulse rate was 88 beats per minute, regular, with good volume with a blood pressure of 100/60mmHg. His respiratory rate was 14 cycles per minute with no flaring of alae nares, and no intercostal nor subcostal recessions. There were no chest wall deformities or tenderness; air entry was reduced in right lower zones; breath sounds were however vesicular with no added sounds. His abdomen was full, soft, non-tender, with no organomegaly and shifting dullness. His central nervous system was grossly normal.

His initial Random Blood Sugar (RBS) (point of care testing) was 23.9mmol/l and a urine routine examination revealed urine glucose of 3+ and urine ketones of 3+. His electrocardiogram (ECG) showed Sinus Tachycardia with a heart rate of 115 beats per minutes, and ST-depression in anterior-lateral leads i.e., leads I, aVL, and V3 to V6 which was suggestive of Acute Myocardial Ischaemia. There was also Left Axis Deviation (LAD) with Left Bundle Branch Block (LBBB).

The following differential diagnoses were entertained:

1. Diabetic Ketoacidosis
2. Myocardial Infarction
3. Chest infection

Management was initiated with modified Alberti's regimen^15^ using soluble insulin and resuscitation with normal saline. Other medications administered included parenteral morphine 10mg, soluble Aspirin 150mg stat then 75mg daily, lisinopril 2.5mg daily, isosorbide dinitrate 10mg tds, atorvastatin 20mg nocte, IV Amoxicillin/clavulanic acid 1.2g 8hourly, oral azithromycin 500mg daily for 3 days and Laboratory investigations were requested. The initial chest X-ray was reported as normal by a radiologist and no further imaging was done. His laboratory investigation results during the course of his admission are shown in Table 1

**Table 1:** Results of laboratory tests during admission

Laboratory Test	Result
**Initial RBS**	23.9mmol/l
**Haemoglobin**	13.3g/dl
**Urine glucose**	3+
**Urine ketones**	3+
**Urine protein**	negative
**S-Sodium**	135mmol/l (136–145)
**Corrected S- Sodium**	140.3mmol/l
**S-Potassium**	4.2 mmol/l (3.5–5.1)
**S-Chloride**	98mmol/l (98–107)
**S-CO_2_**	28.91mmol/l (22–29)
**S-Urea**	6.8 mmol/l (2.1–7.1)
**S-Creatinine**	90 mmol/l (62–106)
**eGFR (CKD-EPI)**	83 ml/min
**C-Troponin T**	3.770 mg/ml (0.0–0.014)
**C-Troponin I**	18.38 ng/ml (<0.16)
**S-Cholesterol**	5.0 mmol/l
**S-LDL cholesterol**	3.3 mmol/L
**HDL cholesterol**	1.3 mmol/l
**Non-HDL cholesterol**	3.7 mmol/l
**All other cholesterol parameters were normal**	
**Chest-Xray**	Normal Chest Radiographs
**ECG**	Sinus Tachycardia, ST depression in anterior-lateral leads, I, aVL, V3 – V6, LAD with LBBB
**SARS-COV-2 VIRUS**	Positive

Despite treatment, he suddenly desaturated with his peripheral oxygen saturation dropping below 85% on room air on the second day of admission. However, his average blood glucose on the second day had decreased to 12.4mmol/l. Intranasal oxygen was commenced at 5 litres/min and subcutaneous enoxaparin 80mg 12hourly was added. The patient was kept in isolation with the suspicion of COVID-19. Samples were immediately taken for COVID-19 testing.

The hyperglycaemia improved significantly with the Alberti's regimen. He was switched to sliding scale on the second day and maintenance potassium administered.

He was then continued on subcutaneous pre-mixed insulin regimen on the fourth day. On the fifth day of admission, the COVID-19 test result came back as positive. Subsequently, supportive therapy with oral zinc 10mg twice daily, and oral vitamin C 1g daily were added to his medications. The municipal health directorate was notified for possible transfer to a designated COVID-19 treatment center. Good glycaemic control was maintained with subcutaneous insulin and dietary management.

On day six of admission, the patient had markedly improved, and his saturation was normal (SPO_2_ of 98–99% on room air). The presenting symptoms had resolved and upon consultation with the municipal health directorate COVID-19 response team, the patient was discharged and instructed to self-isolate at home. In view of the fact that the patient admitted to poor adherence to drug therapy prior to his admission, he was discharged on his preadmission medications. He was put on Metformin 1g bid and glibenclamide 5mg bid. In our setting, sulphonylureas are used as second line agents as cost is a major limiting factor in choice of medications. A subsequent cardiology review was arranged. He has since recovered and is well.

Patient signed a written consent for his case to be submitted for publication.

## Discussion

This case report highlights an atypical presentation of COVID-19 in a 55-year old male with diabetes in an urban primary care facility. He presented without the classic symptoms of fever and a flu-like illness. Similar to our case, 70% of a cohort from New York did not have fever.^16^ His initial chest x-ray was normal. Initial radiographs may be normal in COVID-19. Features of COVID-19 pneumonia are often noted later in the course of the disease with most severe imaging findings being noted around 10–12 days. In cases of atypical presentation, a high index of suspicion is needed. Where there is community spread, contact history may be negative. COVID-19 can lead to fluctuations in glucose control, diabetic ketoacidosis and poor outcomes. It can also unmask latent diabetes.^16^

Hyperglycaemia remains a strong prognostic indicator of hospitalized patients with diabetes and COVID-19 infection. Positive outcomes in such patients can be attained through stringent glycaemic control. Although our patient's glucose readily improved with treatment, it is important to mention that co-morbid COVID-19 can make treating hyperglycaemia difficult, requiring parenteral insulin and an overall aggressive approach.

Dipeptidyl peptidase 4 inhibitors, glucagon-like peptide 1 receptor agonists and insulin therapy are the preferred options for hospitalized patients with COVID-19.^18^

In the acute setting, medications such as sodium glucose co-transporter-2 inhibitors (SGLT 2 inhibitors) and metformin must be discontinued. SGLT 2 inhibitors are associated with an increased risk of euglycaemic ketoacidosis and metformin is associated with an increased risk of lactic acidosis in renal impairment. Use of continuous intravenous insulin infusion is effective for maintaining glycaemic control to improve health outcomes in patients with COVID-19.^17^,^18^

Persons living with diabetes may experience the impact of COVID-19 on their health through disruptions in their diet plans and physical activity, coupled with an increase in stress and mental health challenges.^17^ Opportunities for exercise are currently limited due to the restrictions imposed by governments to help curb the spread of the virus. Access to fresh fruits and vegetables may be limited and patients may have challenges in purchasing insulin.^18^ These factors may result in poor glycaemic control with resulting complications such as diabetic ketoacidosis and acute myocardial infarction.

Doctors working in low resource primary care facilities see undifferentiated cases and often have access to limited laboratory investigations resulting in delays in the diagnosis of COVID-19. Diagnosis in such low resource settings is heavily dependent on a good history and physical examination findings. A high index of suspicion is required in the setting of community spread of COVID-19. The patient had been diagnosed with diabetes five years prior to presentation. He presented with hyperglycaemia. During admission, his oxygen saturation which had been normal on presentation, dropped suddenly. He had no history of hypertension which was evident by the normal blood pressure recorded at presentation and throughout admission.

## Conclusion

This case highlights the need for a high index of suspicion for COVID-19 in cases of significant hyperglycaemia and sudden desaturation in persons known to have diabetes. A high index of suspicion at the frontline will facilitate early identification, notification for testing, isolation, treatment, contact tracing and possible referral or coordination of care with other specialists. Early identification will protect healthcare workers and patients alike from cross-infection.
